# Genetic Parameters for Limousine Interbeef Genetic Evaluation of Calving Traits

**DOI:** 10.3390/genes15020216

**Published:** 2024-02-07

**Authors:** Zdeňka Veselá, Michaela Brzáková, Alexandra Novotná, Luboš Vostrý

**Affiliations:** 1 Department of Genetics and Breeding of Farm Animals, Institute of Animal Science, 104 00 Prague, Czech Republic; mbrzakova781@gmail.com (M.B.); novotna.alexandra@vuzv.cz (A.N.); vostry@af.czu.cz (L.V.); 2 Faculty of Agrobiology, Food and Natural Resources, Czech University of Life Sciences Prague, 165 00 Prague, Czech Republic

**Keywords:** Interbeef, beef cattle, calving traits, international genetic evaluation, bending

## Abstract

The aim of this study was to estimate across-country genetic correlations for calving traits (birth weight, calving ease) in the Limousine breed. Correlations were estimated for eight populations (Czech Republic, joint population of Denmark, Finland, and Sweden, France, Great Britain, Ireland, Slovenia, Switzerland, and Estonia). An animal model on raw performance accounting for across-country interactions (AMACI) was used. (Co)variance components were estimated for pairwise combinations of countries. Fixed and random effects were defined by each country according to its national genetic evaluation system. The average across-country genetic correlation for the direct genetic effect was 0.85 for birth weight (0.69–0.96) and 0.75 for calving ease (0.62–0.94). The average correlation for the maternal genetic effect was 0.57 for birth weight and 0.61 for calving ease. After the estimation of genetic parameters, the weighted bending procedure was used to compute the full Interbeef genetic correlation matrix. After bending, direct genetic correlations ranged from 0.62 to 0.84 (with an average of 0.73) for birth weight and from 0.58 to 0.82 (with an average of 0.68) for calving ease.

## 1. Introduction

Efforts to create an international genetic evaluation for beef cattle started in 2001 with the EUropean BEef EVALuation project (EUBEEVAL) [1]. Renand et al. [2] explored the presence of genotype–environment interactions, estimated genetic correlations for weaning weight measured in France, Great Britain, and Ireland, and highlighted the need for an international evaluation. Phocas et al. [3,4] compared three models for beef cattle international evaluation. An animal model on raw performance, accounting for heterogeneous variance and different genetic correlations between countries (AMACI—animal model accounting for across-country interactions), was the model of choice for obtaining the best consistency between national and international ranking of animals and the best estimates of genetic parameters across countries. This model allows the prediction of international breeding values for all animals: sires, dams, and calves. In 2007, ICAR established a new service called Interbeef [5]. Venot et al. [6] performed the first pilot study and estimated across-country genetic correlations for weaning weight between France, Ireland, and the United Kingdom for the Limousine breed. Three years later, genetic correlations were estimated for France, Ireland, the United Kingdom, Sweden, and Denmark for the Charolais and Limousine breeds [7]. Since then, Interbeef has extended its scope and now provides services for five breeds (Charolais, Limousine, Beef Simmental, Aberdeen Angus, and Hereford), 14 countries (Australia, the Czech Republic, Denmark, Estonia, Finland, France, Germany, Ireland, Italy, Latvia, Slovenia, Sweden, Switzerland, and the United Kingdom), and three traits (age-adjusted weaning weight [5], and birth weight and calving ease grouped as calving traits [8,9]).

Genetic correlations (r_g_) are key for international evaluations, as they determine how much the information about relatives recorded in one country affects the animal’s international breeding value in another country [10]. The estimation of across-country r_g_ in beef cattle is challenging due to the usually lower level of genetic connectedness, related to the lower usage of AI, compared to dairy breeds like Holstein [11] and the need to incorporate maternal genetic effects and maternal permanent environmental effects into the model [2,4]. Therefore, the estimation of across-country r_g_ is highly computationally demanding and time-consuming. Bonifazi et al. [12] investigated and compared the impact of strategies of sub-setting on estimated across-country r_g_ and their computational requirements. Using data sub-setting strategies reduced the required computing time to 22% of that required when using all data. Reducing the data mainly affected the estimates of direct–maternal within-country and between-country r_g_ but had a small impact on the estimated direct and maternal across-country r_g_. However, given the need to estimate across-country r_g_ for new countries in a reasonably short time, a bivariate approach combined with the sub-setting of data is a common practice in Interbeef [9,13]. The objective of this study was to estimate across-country genetic correlation for calving traits (birth weight, calving ease) in the Limousine breed.

## 2. Materials and Methods

### 2.1. Data

Anonymized phenotypic and pedigree data were provided by Interbull in 2022. Eight populations participated in the calving traits project: Czech Republic (CZE), Denmark, Finland, and Sweden as one joint population (DFS), France (FRA), Great Britain (GBR), Ireland (IRL), Slovenia (SVN), Estonia (EST), and Switzerland (CHE). Ireland provided performances for purebred and crossbred animals as well, whereas all other countries participated with purebred animals only. However, not all populations participated in all breed/trait combinations (Table 1).

The definition of birth weight (BWT) performance was the same in all countries. The definition of calving ease (CAE) was different and based on national evaluation practices (Supplementary Table S1) as follows: (i) three points (CHE—100, 200, and 300; EST—1, 2, and 3); (ii) four points, 1–4 (CZE, IRL); (iii) five points, 1–5 (FRA, GBR, SVN); (iv) four points (1 for easy) corrected for heterogeneous variance in different countries (DFS; therefore, the data provided by DFS were decimals instead of integers). Most countries scored CAE from the easiest to the most complicated birth, except Switzerland, which used a reverse scale (100 for hard pull, 200 for easy pull, and 300 for no assistance).

### 2.2. Data Edits

Each country uploaded phenotypic performances edited according to its national evaluation standard. An appropriate data structure and connectedness must be ensured for genetic parameter estimation. Therefore, we had to perform subsequent data editing. Embryo transfer calves, calves without a known sire and maternal grandsire (MGS), contemporary groups (CG) with only one sire, CGs with fewer than three animals with records, and herds with no variation in CAE scores or BWT were removed (Table 2 and Table 3).

After that, performance data files were prepared for pairwise country genetic parameter estimation according to the genetic connection between countries. The sequence of conditions was applied differently according to the population size to achieve the optimal size and structure of a dataset for variance components estimation. Only calves born since the year 2006 (2010 for FRA) were kept, a minimum size of CG was applied (3 for small, 5 for medium, and 20 for large populations—FRA), a minimum number of calves per dam was set (2 for small, 3 for medium, and 4 for large populations), and a minimum number of sires per CG was set (2 for small, 3 for medium, and 4 for large populations). To maintain connections between countries, only CGs with at least one calf connected through a common sire, a grandsire, or a grand-grandsire were used. All CGs were used for less numerous populations (EST, SVN, and IRL for BWT). For medium and large populations, the only CGs that were used were those with a higher percentage of calves connected through common sires and grandsires in three generations—10% for CZE, 30% for CHE, 40% for DFS, GBR, and IRL (for CAE), and 90% for FRA (Table 4).

A pedigree file was built for each pairwise combination and contained five generations without a phantom parent group. The sizes of the performance files and pedigrees for each pairwise combination of countries are presented in Supplementary Table S2 (BWT) and Table S3 (CAE).

### 2.3. Model

An animal model on raw performance accounting for across-country interactions (AMACI) was used [4,12]. (Co)variance components were estimated by a single-trait (CAE and BWT separately) animal model using the average information REML (AIREML) of the blupf90+ family programs [14,15] for pairwise combinations of countries [4]. Fixed and random effects were defined by each country according to its national genetic evaluation system [16,17,18,19,20,21,22,23,24,25,26] as:

**y** = **Xb** + **Cr** + **Zu** + **Wm** + **Pp** + **e** (1)

Since the full model with direct and maternal additive genetic effects did not converge for some pairwise combinations of countries, the model without maternal additive genetic effects was run in these cases:

**y** = **Xb** + **Cr** + **Zu** + **Pp** + **e** (2)

where **y** is the vector of performances ordered by country **y**′ = (**y**_1′_, **y**_2′_); **b** is the vector of fixed effects (CZE—sex-twinning, age at calving, year; DFS—contemporary group, sex, age at calving, season; FRA—contemporary group, sex, parity-age at calving, season; GBR—herd-year, birth month, birth type, sex for BWT, age of dam for BWT, parity-sex of calf for CAE; IRL—sex, parity, age at calving, year; fixed regression of breed composition for animal, fixed regression of breed composition for dam of animal, fixed regression of specific heterosis; SVN—contemporary group, sex, parity, herd-year; EST—herd-year, season, sex-twinning, age at calving, year; CHE—age at calving, sex, year-season, parity-age at calving, breed composition); **r** is the vector of random environmental effects (CZE—contemporary group; IRL—herd-year-season; CHE—herd-year); **u** is the vector of random direct additive genetic effects (for all countries); **m** is the vector of random maternal additive genetic effects (for CZE, DFS, FRA, GBR, SVN, and CHE); **p** is the vector of random maternal permanent environmental effects (for CZE, DFS, FRA, GBR, ILR, and CHE); **X** and **C** are incidence matrices linking records to fixed (**b**) and random (**r**) environmental effects; **Z**, **W**, and **P** are incidence matrices relating **u**, **m**, and **p** to **y**; and **e** is the vector of random residual effects.

We assumed that

$$var\left(r\right)=\left[\begin{array}{cc} \sigma_{r_{1}}^{2} & 0\\ 0 & \sigma_{r_{2}}^{2} \end{array}\right],$$

$$var\left(p\right)=\left[\begin{array}{cc} \sigma_{p_{1}}^{2} & 0\\ 0 & \sigma_{p_{2}}^{2} \end{array}\right],$$

$$var\left(e\right)=\left[\begin{array}{cc} \sigma_{e_{1}}^{2} & 0\\ 0 & \sigma_{e_{2}}^{2} \end{array}\right]$$

For the model with direct and maternal additive genetic effects

$$var\left[\begin{array}{c} u_{1}\\ u_{2}\\ m_{1}\\ m_{2} \end{array}\right]=\left[\begin{array}{cccc} \sigma_{u_{1}}^{2} & \sigma_{u_{1},u_{2}} & \sigma_{u_{1},m_{1}} & 0\\ \sigma_{u_{1},u_{2}} & \sigma_{u_{2}}^{2} & 0 & \sigma_{u_{2},m_{2}}\\ \sigma_{u_{1},m_{1}} & 0 & \sigma_{m_{1}}^{2} & \sigma_{m_{1},m_{2}}\\ 0 & \sigma_{u_{2},m_{2}} & \sigma_{m_{1},m_{2}} & \sigma_{m_{2}}^{2} \end{array}\right]⊗A$$

For the model without maternal additive genetic effects

$$var\left[\begin{array}{c} u_{1}\\ u_{2} \end{array}\right]=\left[\begin{array}{cc} \sigma_{u_{1}}^{2} & \sigma_{u_{1},u_{2}}\\ \sigma_{u_{1},u_{2}} & \sigma_{u_{2}}^{2} \end{array}\right]⊗A$$

where $\sigma_{u_{i}}^{2}$ is the direct additive genetic variance for population *i*; $\sigma_{m_{i}}^{2}$ is the maternal additive genetic variance for population *i*; $\sigma_{u_{i},u_{j}}^{2}$ is the direct additive genetic covariance between populations *i* and *j*; $\sigma_{m_{i},m_{j}}^{2}$ is the maternal additive genetic covariance between populations *i* and *j*; $\sigma_{u_{i},m_{i}}^{2}$ is the additive genetic covariance between the direct and the maternal genetic effect for population *i*; $\sigma_{r_{i}}^{2}$ is random environmental effect for population *i*; $\sigma_{p_{i}}^{2}$ is the permanent environmental variance for population *i*; $\sigma_{e_{i}}^{2}$ is the residual error variance for population *i*; and **A** is the relationship matrix. We expected that additive genetic covariances between the direct genetic effect for population *i* and the maternal genetic effects for population *j* ($\sigma_{u_{i},m_{j}}^{2})$ would be near 0; therefore, these were fixed to 0 to help with convergence of estimation.

After the estimation of genetic parameters, the weighted bending procedure described by Jorjani et al. (2003) [27] with a threshold value of 10^−4^ was used to compute the full Interbeef genetic correlation matrix in three steps, as follows:

1. Direct genetic correlations which were not statistically significant were set to average values with a standard error of 0.3. The matrix of direct genetic correlations was then bended, with standard errors used as weights.
2. Maternal genetic correlations which were not statistically significant or for which the estimation did not converge were set to a value of 0.6 and a standard error of 0.4. The matrix of maternal genetic correlations was then bended, with standard errors used as weight.
3. A full matrix of direct and maternal genetic correlations for both calving traits was created with within-country correlations provided by the participating countries (Table 5) [25]. Across-country correlations between direct and maternal effects and direct and maternal correlations between BWT and CAE were set to 0. The full correlation matrix was then bended with a weighting factor equal to the reciprocal of 1000 plus the number of common sires multiplied by 20 for direct correlations, 500 plus the number of common maternal grandsires multiplied by 5 for maternal correlations, the number 1000 for non-converged or statistically not significant direct correlations, the number 500 for non-converged or statistically not significant maternal correlations, the number 9999 for non-zero within-country correlations, and the number 1 for direct–maternal and BWT–CAE correlations between countries. These weighting factors aimed to keep the within-country genetic correlations provided by the participating countries without significant changes and minimize changes in the estimated across-country correlations. Therefore, the highest values were set for within-country genetic correlations, and slightly lower values were set for across-country correlations within direct and maternal genetic effects. The lowest values were set for across-country genetic correlations between direct and maternal effects and between BWT and CAE. Weighted bending minimizes the changes to more reliable estimates at the expense of larger changes to less reliable ones [27]. The matrix of weights used for bending the full Interbeef correlation matrix is presented in Supplementary Table S4.

After that, a full genetic (co)variance matrix used to predict international breeding values was constructed using within-country genetic variances (Table 6).

## 3. Results

### 3.1. Connectedness

The total number of bulls used within countries, the number of common bulls, and the number of common maternal grandsires are presented in Table 7 (BWT) and Table 8 (CAE). The total number of bulls within countries corresponded to the population size and ranged from 177 in SVN to 89,153 in FRA. The number of common bulls varied from 13 for CHE–SVN to 388 for FRA–GBR for BWT and from 3 for EST–SVN to 402 for FRA–GBR for CAE. The highest numbers of common bulls were found for a combination of France with other countries. The number of common maternal grandsires was higher than the number of common bulls and varied from 16 for IRL–SVN to 1384 for FRA–GBR for BWT, and from 9 for EST–SVN to 1084 for FRA–GBR for CAE. The highest numbers of common bulls were recorded for FRA–GBR (388 BWT, 402 CAE), GBR–IRL (172 BWT, 309 CAE), CZE–FRA (262 BWT, 262 CAE), FRA–IRL (118 BWT, 230 CAE), and DFS–FRA (224 BWT, 216 CAE).

The total number of common bulls was 2238 for BWT and 2635 for CAE. The distribution of common bulls according to the number of connected populations is presented in Table 9 and Table 10. In the dataset of BWT performances, about 55.4% of common bulls connected two populations, and 18.8, 11.3, 7.6, 5.1, and 1.9% connected from three to seven populations. In the dataset of CAE performances, about 51.8% of common bulls connected two populations, and 19.5, 11.6, 6.4, 6.8, and 4.0% connected from three to seven populations. The majority of common bulls were from France (82.6% for BWT and 78.4% for CAE), followed by Great Britain (9.2% for BWT and 10,0% for CAE), Germany (4.1% for BWT and 3.9% for CAE), Ireland (1.6% for BWT and 2.9% for CAE), and Denmark (1.4% for BWT and 2.9% for CAE).

The percentage of offspring directly connected through common sires between two populations is presented in Table 11 and Table 12. For this analysis, only calves born from 2006 to 2021 were used to secure the same period for all populations. The highest average percentage of offspring of common sires was in the population from the Czech Republic (12.39% for BWT and 11.91% for CAE), followed by France (9.4% for BWT and 9.27% for CAE), Ireland (9.61% for BWT and 3.34% for CAE), Great Britain (7.47% for BWT and 6.75% for CAE), Switzerland (6.65% for BWT and 6.32% for CAE), DFS (3.26% for BWT and 2.86% for CAE), Slovenia (2.6% for BWT and 2.18% for CAE), and Estonia (1.54% for CAE). Here, it is necessary to mention that Ireland sent data not only from purebred but also from crossbred animals, which is the reason for the lower percentage connectedness in this population. The most important connected population was that from France, with which the other populations were connected through 11.21% of offspring on average for BWT and 8.78% for CAE. On average, the percentage of common offspring through all populations was slightly higher for BWT (7.34%) than for CAE (5.52%). Finally, there were some highly connected populations. For BWT, they were CZE–FRA (21.14% of calves from CZE were directly connected through common sires with FRA calves), followed by IRL–GBR (20.18%), GBR–IRL (16.63%), CHE–FRA (14.59%), and CZE–DFS (14.53%). For CAE, they were CZE–FRA (21.14%), GBR–IRL (21.3%), CZE–DFS (14.43%), and CHE–FRA (13.85%).

### 3.2. Across-Country Genetic Correlations

The estimated across-country genetic correlations and standard errors for BWT and CAE are presented in Table 13 and Table 14, respectively. The average across-country genetic correlation for the direct genetic effect was 0.85 for BWT (ranging from 0.69 for CZE–CHE to 0.96 for IRL–CHE) and 0.75 for CAE (ranging from 0.62 for CZE–FRA to 0.94 for IRL–EST). The average correlation for maternal genetic effect was 0.57 for BWT and 0.61 for calving ease. For CHE, the direct genetic correlations for CAE were negative, since CHE used a reverse scale compared to other countries. Many maternal genetic correlations were not statistically significant, or the estimation did not converge. Some direct genetic correlations for small populations with lower connectedness to other countries, such as Slovenia, Estonia, and Switzerland, were not statistically significant. In these cases, the lack of connectedness complicated the estimation of genetic correlations. Direct genetic correlations were lower for CAE than for BWT.

### 3.3. Interbeef Correlation Matrix

**Figure 1:**
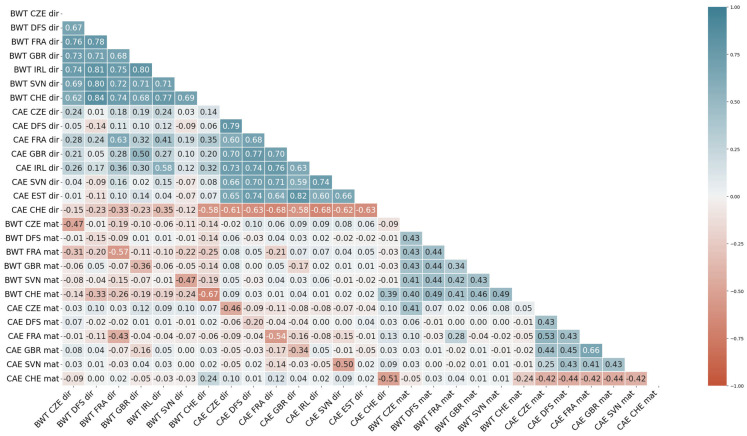
Full Interbeef correlation matrix after bending. CZE—Czech Republic; DFS—Denmark, Finland, and Sweden; FRA—France; GBR—Great Britain; IRL—Ireland; SVN—Slovenia; EST—Estonia; CHE—Switzerland. BWT—birth weight; CAE—calving ease; dir—direct effect; mat—maternal effect.

The full Interbeef genetic correlation matrix before bending is presented in Supplementary Table S5. The full Interbeef genetic correlation matrix after bending is presented in Figure 1. To achieve a positive definite matrix, 41,934 iterations of bending were needed. The average direct genetic correlation after bending was 0.73 for BWT (ranging from 0.62 for CZE–CHE to 0.84 for DFS–CHE) and 0.68 for CAE (ranging from −0.58 for GBR–CHE to 0.82 for GBR–EST). The average maternal genetic correlation was 0.43 for BWT and 0.44 for CAE. A full matrix with genetic correlations and genetic (co)variances is presented in Supplementary Table S6.

The differences in the genetic correlations after bending and before bending are presented in Supplementary Table S7. The direct genetic correlations for BWT were on average about 0.1 lower than the genetic correlations before bending. The direct genetic correlations for CAE were on average about 0.08 lower (resp. higher for CHE) than the genetic correlations before bending. The highest differences were for small populations with a lower number of common bulls, such as those of Slovenia (on average, −0.14 for BWT and −0.10 for CAE) and Switzerland (on average, −0.12 for BWT and −0.10 for CAE). Within-country correlations differed only slightly (from −0.06 to 0.05).

## 4. Discussion

In this study, we estimated genetic correlations between participating countries in the Interbeef international genetic evaluation.

The estimation of across-country r_g_ in beef cattle is challenging due to an usually lower level of genetic connectedness related to the lower usage of AI compared to dairy breeds like Holstein [11]. The number of common bulls presented in our study is consistent with those in previous studies [7,10,12,13,28]. Moreover, 55.4% (BWT) and 51.8% (CAE) of common bulls connected only two populations. Bonifazi et al. [12] stated that an even higher percentage (73.4%) of common limousine bulls connected only two populations in the performance set for weaning weight. The majority of common bulls in our study were from France (82.6% for BWT and 78.4% for CAE), which corresponds to 85.5% of common bulls from France in the study on weaning weight by Bonifazi et al. [12]. Bouquet et al. [29] analyzed the genetic structure of the European Charolais and Limousine breeds and pointed out that most of the founder genes derived from the French population. For the countries currently participating in Interbeef, the across-country connection in many cases hinged on linked French sires [13]. Although some populations (such as those of Estonia and Slovenia) in our study had a small number of connecting common bulls, there were no missing connections between populations. A similar limited connection was reported for the Charolais breed [7,13]. However, for both these breeds, the participation of the French population provides acceptable connectedness through all data, which is sufficient for the estimation of genetic correlations.

The estimated direct genetic correlations in our study ranged from 0.69 to 0.96 (with an average of 0.85) for BWT and from 0.62 to 0.94 (with an average of 0.75) for CAE. Lower genetic correlations for CAE corresponded to lower heritability coefficients (Table 7). Some estimations of direct genetic correlations for small populations with low connectedness to other populations (such as those of Slovenia, Estonia, and Switzerland) were not statistically significant. The estimation of maternal genetic correlations using the full model with direct and maternal genetic effects was more difficult, and many maternal genetic correlations were not statistically significant, or the estimation failed to converge. In these cases, the model with direct genetic effect and without maternal genetic effects was run. The same difficulties in the estimation of maternal genetic correlations across countries for weaning weight in the Limousine and Charolais breeds were reported in previous studies [7,13] and in an international evaluation of weakly linked dairy populations [30,31]. In the case of these breeds, the estimation of maternal genetic correlations is difficult because of a lower exchange of bulls used for breeding females compared to bulls used as terminal sires [13].

The bending method by Jorjani et al. [27,32] was applied to obtain a full definite positive matrix. This approach is used in cases with a single correlation and a covariance matrix that is constructed from separately estimated elements, for example, in the international genetic evaluation of dairy cattle using the MACE methodology [33]. This method takes into account the amount of information available for the estimation [7]. Standard errors of estimation in combination with the number of common bulls between the countries were used to perform the weighted bending of the full Interbeef correlation matrix in our study. A similar approach was used for the construction of a full Interbeef correlation matrix for weaning weights [7,13]. The direct genetic correlations after bending were lower than the estimated direct genetic correlations from the pairwise model. The direct genetic correlations ranged from 0.62 to 0.84 (with an average of 0.73) for BWT and from −0.58 to 0.82 (with an average of 0.68) for CAE, which is in accordance with previous studies on Interbeef genetic correlations for weaning traits. The genetic correlations estimated from pairwise combinations of two countries [7] were higher than the genetic correlations estimated using all populations in one estimation together [12]. The use of weights in the weighted bending procedure secures that genetic correlations for the combination of populations with strong connections through common sires and larger size change less at each bending iteration compared to those with weak connections.

The disadvantages of the bending procedure could be overcome by using a multivariate model that includes all the populations simultaneously in one estimation [12]. However, this approach has some disadvantages as well. First, it is time-consuming and highly computationally demanding. Bonifazi et al. [12] compared different sub-setting strategies in the estimation of across-country genetic correlations for weaning weight in the Limousine breed. Using all the data, it took 43 days and 23 h to estimate all across-country genetic correlations. The data sub-setting scenarios decreased the total computing time by 9 to 16 days. However, such estimation would be much more complicated in the case of calving traits, since we worked with two correlated traits (BWT and CAE).

The bending approach has some benefits as well. Bending the genetic correlation matrix allows one to keep national variances unchanged, which is desired in an international evaluation [27], since countries can use a more representative national dataset for variance components estimation [12]. Finally, from a practical point of view, the bending approach enables the estimation of genetic correlations for a new country (population) joining Interbeef separately, without the need to run the time-consuming multivariate model including all populations. This enables the construction of an updated (co)variance matrix within the time frame for a new VCE estimation of the Interbeef test run, which is currently 20 days [34].

## 5. Conclusions

Across-country genetic correlations for the pairwise combination of eight populations of the Limousine breed for two calving traits (birth weight and calving ease) were estimated by using AIREML. After that, the weighted bending procedure was applied, and a full (co)variance matrix for the international genetic evaluation of calving traits in the Limousine breed was constructed.

## Figures and Tables

**Table 1:** Number of records in the performance files.

	BWT			CAE		
Population	N	%	Year of Birth	N	%	Year of Birth
CZE	25,792	0.40	1991–2021	25,792	0.33	1991–2021
DFS	177,946	2.76	1998–2021	318,954	4.10	1998–2021
FRA	5,964,620	92.35	1963–2021	5,930,554	76.17	1965–2021
GBR	212,787	3.29	1972–2021	176,132	2.26	1989–2021
IRL	19,300	0.30	1998–2022	1,245,339	15.99	1950–2022
SVN	3103	0.05	1995–2021	3877	0.05	2004–2021
EST	-	-	-	24,306	0.31	1999–2022
CHE	55,308	0.86	2006–2022	61,020	0.78	2006–2022
Total	6,458,856			7,785,974		

CZE—Czech Republic; DFS—Denmark, Finland, and Sweden; FRA—France; GBR—Great Britain; IRL—Ireland; SVN—Slovenia; EST—Estonia; CHE—Switzerland.

**Table 2:** Number of animals removed from the performance data files (BWT).

Population	ET	Sire	CG	SireCG	NoVar	After Edits
CZE	0	425	533	2071	86	22,677
DFS	294	23,519	1281	33,833	2065	116,954
FRA	0	1,677,531	49,925	262,256	3927	3,970,981
GBR	9668	5581	9870	27,365	765	159,538
IRL	0	5309	861	3395	538	9197
SVN	0	210	0	0	0	2893
CHE	0	3141	3717	4649	252	43,549

CZE—Czech Republic; DFS—Denmark, Finland, and Sweden; FRA—France; GBR—Great Britain; IRL—Ireland; SVN—Slovenia; CHE—Switzerland. ET—calves from embryo transfer; Sire—unknown sire or MGS; CG—CGs with fewer than three animals; SireCG—CGs with only one sire; NoVar—herds with no variation.

**Table 3:** Number of animals removed from the performance data files (CAE).

Population	ET	Sire	CG	SireCG	NoVar	After Edits
CZE	0	302	402	1306	4236	19,546
DFS	105	62,659	4375	32,194	99,244	120,377
FRA	0	1,587,609	42,405	239,048	156,332	3,905,160
GBR	6654	4056	3411	16,163	3009	142,839
IRL	0	642,833	67,048	243,469	113,655	178,334
SVN	0	101	13	4	714	3045
EST	0	847	106	4335	4946	14,072
CHE	0	3099	3223	4472	7617	42,609

CZE—Czech Republic; DFS—Denmark, Finland, and Sweden; FRA—France; GBR—Great Britain; IRL—Ireland; SVN—Slovenia; EST—Estonia; CHE—Switzerland. ET—calves from embryo transfer; Sire—unknown sire or MGS; CG—CGs with fewer than three animals; SireCG—CGs with only one sire; NoVar—herds with no variation.

**Table 4:** Conditions for the preparation of the performance data files for variance components estimation.

Population	YoB	CG	Dam	SireCG	Conn
CZE	2006	5	3	3	10%
DFS	2006	5	3	3	40%
FRA	2010	20	4	4	90%
GBR	2006	5	3	3	40%
IRL	2006	3 (BWT) 5 (CAE)	2 (BWT) 3 (CAE)	2 (BWT) 3 (CAE)	All (BWT) 40% (CAE)
SVN	2006	3	2	2	All
EST	2006	3	2	2	All
CHE	2006	5	3	3	30%

CZE—Czech Republic; DFS—Denmark, Finland, and Sweden; FRA—France; GBR—Great Britain; IRL—Ireland; SVN—Slovenia; EST—Estonia; CHE—Switzerland. BWT—birth weight; CAE—calving ease. YoB—year of birth; CG—minimum size of contemporary group; Dam—minimum number of offspring per dam; SireCG—minimum number of sires per contemporary group; Conn—minimum percentage of calves connected through common sires and grandsires in three generations.

**Table 5:** Within-country genetic correlations used to construct a full Interbeef correlation matrix [25].

	CZE	DFS	FRA	GBR	IRL	SVN	EST	CHE
r_g(dir bwt, dir cae)_	0.25	0	0.69	0.53	0.62	0	-	−0.63
r_g(mat bwt, mat cae)_	0.42	0	0.28	0	-	0	-	−0.24
r_g(dir bwt, mat bwt)_	−0.48	−0.15	−0.61	−0.37	-	−0.49	-	−0.72
r_g(dir cae, mat cae)_	−0.47	−0.2	−0.56	−0.35	-	−0.51	-	−0.53
r_g(dir bwt, mat cae)_	0.04	0	−0.45	0	-	0	-	0.24
r_g(mat bwt, dir cae)_	−0.01	0	−0.20	0	-	0	-	0.39

CZE—Czech Republic; DFS—Denmark, Finland, and Sweden; FRA—France; GBR—Great Britain; IRL—Ireland; SVN—Slovenia; EST—Estonia; CHE—Switzerland. r_g_—genetic correlation; dir—direct genetic effect; mat—maternal genetic effect; bwt—birth weight; cae—calving ease.

**Table 6:** Within-country genetic variances used to construct a full genetic (co)variance matrix and coefficients of heritability [25].

	CZE	DFS	FRA	GBR	IRL	SVN	EST	CHE
σ^2^_dir bwt_	4.1	8.7689	4.985	3.6	7.3139	11.265	-	12.126
σ^2^_mat bwt_	0.9	2.3712	1.08	0.67	-	4.321	-	2.093
σ^2^_dir cae_	0.0169	0.0116	0.0041	0.04	0.0788	0.0294	0.0045	287.256
σ^2^_mat cae_	0.0033	0.0059	0.0013	0.02	-	0.0073	-	149.438
h^2^_dir bwt_	0.21	0.38	0.43	0.30	0.14	0.39	-	0.47
h^2^_mat bwt_	0.05	0.10	0.09	0.06	-	0.15	-	0.08
h^2^_dir cae_	0.17	0.04	0.05	0.11	0.14	0.10	0.10	0.17
h^2^_mat cae_	0.03	0.02	0.02	0.06	-	0.02	-	0.09

CZE—Czech Republic; DFS—Denmark, Finland, and Sweden; FRA—France; GBR—Great Britain; IRL—Ireland; SVN—Slovenia; EST—Estonia; CHE—Switzerland. σ^2^—genetic variance; h^2^—coefficient of heritability; dir—direct genetic effect; mat—maternal genetic effect; bwt—birth weight; cae—calving ease.

**Table 7:** Total number of bulls used within a country (diagonal), number of common bulls (below diagonal), and number of common maternal grandsires (above diagonal) for birth weight.

	CZE	DFS	FRA	GBR	IRL	SVN	CHE
CZE	1018	115	672	127	77	18	111
DFS	103	5489	325	152	83	22	111
FRA	262	224	89,153	1384	148	83	620
GBR	90	119	388	10,695	218	21	111
IRL	73	77	118	172	1124	16	56
SVN	15	24	44	15	14	177	22
CHE	79	83	61	61	44	13	2832

CZE—Czech Republic; DFS—Denmark, Finland, and Sweden; FRA—France; GBR—Great Britain; IRL—Ireland; SVN—Slovenia; CHE—Switzerland.

**Table 8:** Total number of bulls used within a country (diagonal), number of common bulls (below diagonal), and number of common maternal grandsires (above diagonal) for calving ease.

	CZE	DFS	FRA	GBR	IRL	SVN	EST	CHE
CZE	1018	115	672	137	113	18	40	111
DFS	101	7650	311	155	126	24	106	108
FRA	262	216	88,573	1084	473	74	92	619
GBR	106	123	402	9330	487	28	30	116
IRL	97	113	230	309	31,095	22	35	101
SVN	15	21	33	16	18	209	9	25
EST	32	60	53	29	29	3	542	60
CHE	80	83	153	67	66	12	28	3173

CZE—Czech Republic; DFS—Denmark, Finland, and Sweden; FRA—France; GBR—Great Britain; IRL—Ireland; SVN—Slovenia; EST—Estonia; CHE—Switzerland.

**Table 9:** Number of common bulls according to the number of connected populations for birth weight.

	Number of Connected Populations						
COU	2	3	4	5	6	7	Sum
CAN	2	3					5
CHE	6						6
DEU	34	30	12	10	6		92
DNK	26	6					32
FRA	1028	291	224	155	108	42	1848
GBR	114	72	16	5			207
IRL	22	15					37
LUX	4						4
NOR	2						2
SWE		3					3
USA	2						2
Sum	1240	420	252	170	114	42	2238

COU—country of first registration; CAN—Canada; CHE—Switzerland; DEU—Germany; DNK—Denmark; FRA—France; GBR—Great Britain; IRL—Ireland; LUX—Luxemburg; NOR—Norway; SWE—Sweden; USA—United States of America.

**Table 10:** Number of common bulls according to the number of connected populations for calving ease.

	Number of Connected Populations						
COU	2	3	4	5	6	7	Sum
CAN	4	3					7
CHE	8	3					11
CZE	8						8
DEU	36	24	20	15	6		101
DNK	66	6	5				77
FRA	1010	372	260	145	174	105	2066
GBR	154	81	20	10			265
IRL	56	21					77
LUX	4						4
NOR	2						2
SWE	8	3					11
USA	6						6
Sum	1362	513	305	170	180	105	2635

COU—country of first registration; CAN—Canada; CHE—Switzerland; CZE—Czech Republic; DEU—Germany; DNK—Denmark; FRA—France; GBR—Great Britain; IRL—Ireland; LUX—Luxemburg; NOR—Norway; SWE—Sweden; USA—United States of America.

**Table 11:** Percentage of offspring directly connected through common sires between two populations for birth weight.

	Connected Population							
POP	CZE	DFS	FRA	GBR	IRL	SVN	CHE	Mean
CZE		14.53	21.14	12.36	10.76	2.70	12.83	12.39
DFS	4.89		6.21	2.86	2.39	0.52	2.70	3.26
FRA	12.16	12.61		9.63	8.30	2.73	10.97	9.4
GBR	7.91	6.44	10.40		16.63	0.86	2.56	7.47
IRL	12.07	7.90	10.23	20.18		1.16	6.12	9.61
SVN	1.87	2.85	4.69	2.48	1.72		1.99	2.6
CHE	7.43	8.14	14.59	4.91	3.79	1.04		6.65
Mean	7.72	8.74	11.21	8.74	7.26	1.50	6.19	7.34

POP—population for which the percentage of offspring was calculated; CZE—Czech Republic; DFS—Denmark, Finland, and Sweden; FRA—France; GBR—Great Britain; IRL—Ireland; SVN—Slovenia; CHE—Switzerland.

**Table 12:** Percentage of offspring directly connected through common sires between two populations for calving ease.

	Connected Population								
POP	CZE	DFS	FRA	GBR	IRL	SVN	EST	CHE	Mean
CZE		14.43	21.14	13.12	13.66	2.88	5.28	12.85	11.91
DFS	3.91		4.51	2.29	2.23	0.46	4.73	1.91	2.86
FRA	12.17	12.62		10.86	11.10	2.90	4.30	10.97	9.27
GBR	7.45	5.34	9.53		21.30	0.56	0.83	2.24	6.75
IRL	3.33	2.89	4.39	9.94		0.24	0.97	1.65	3.34
SVN	1.83	2.42	4.35	2.01	2.55		0.30	1.83	2.18
EST	1.05	3.84	3.68	0.64	0.60	0.08		0.93	1.54
CHE	7.10	7.96	13.85	5.72	5.76	1.03	2.81		6.32
Mean	5.26	7.07	8.78	6.37	8.17	1.16	2.75	4.63	5.52

POP—population for which the percentage of offspring was calculated; CZE—Czech Republic; DFS—Denmark, Finland, and Sweden; FRA—France; GBR—Great Britain; IRL—Ireland; SVN—Slovenia; EST—Estonia; CHE—Switzerland.

**Table 13:** Estimated across-country genetic correlations for direct effect (below diagonal) and maternal effect (above diagonal), and their standard errors for birth weight.

	**CZE**	**DFS**	**FRA**	**GBR**	**IRL**	**SVN**	**CHE**
CZE		0.33 (0.74) *	0.34 (0.52) *	0.04 (0.45) *	-	nc	0.49 (0.55) *
DFS	**0.70 (0.16)**		0.05 (0.25) *	0.01 (0.31) *	-	0.08 (0.22) *	**0.59 (0.18)**
FRA	**0.95 (0.09)**	**0.88 (0.04)**		**0.41 (0.14)**	-	nc	0.14 (0.63) *
GBR	**0.82 (0.21)**	**0.73 (0.04)**	**0.80 (0.02)**		-	nc	**0.58 (0.17)**
IRL	**0.86 (0.14)**	**0.89 (0.18)**	**0.87 (0.11)**	**0.87 (0.10)**		-	-
SVN	0.56 (0.70) *	**0.89 (0.04)**	**0.87 (0.04)**	0.45 (0.82) *	0.32 (0.63) *		**0.70 (0.21)**
CHE	**0.69 (0.13)**	**0.95 (0.05)**	**0.87 (0.04)**	**0.80 (0.18)**	**0.96 (0.10)**	**0.85 (0.26)**	

CZE—Czech Republic; DFS—Denmark, Finland, and Sweden; FRA—France; GBR—Great Britain; IRL—Ireland; SVN—Slovenia; CHE—Switzerland. nc—Estimation did not converge; *—correlation was not used for the construction of a full Interbeef matrix since it was not statistically significant.

**Table 14:** Estimated across-country genetic correlations for direct effect (below diagonal) and maternal effect (above diagonal), and their standard errors for calving ease.

	CZE	DFS	FRA	GBR	IRL	SVN	EST	CHE
CZE		0.11 (0.36) *	**0.56 (0.14)**	nc	-	0.32 (0.40) *	-	−0.27 (0.77) *
DFS	**0.83 (0.15)**		0.06 (0.33) *	0.13 (0.32) *	-	0.01 (0.25) *	-	0.21 (0.89) *
FRA	**0.62 (0.08)**	**0.70 (0.17)**		**0.67 (0.08)**	-	nc	-	−0.10 (0.69) *
GBR	**0.75 (0.15)**	**0.82 (0.11)**	**0.73 (0.05)**		-	nc	-	−0.41 (0.45) *
IRL	**0.78 (0.23)**	**0.78 (0.22)**	**0.80 (0.13)**	**0.65 (0.15)**		-	-	-
SVN	**0.76 (0.05)**	**0.77 (0.24)**	**0.82 (0.05)**	**0.67 (0.05)**	**0.86 (0.27)**		-	−0.08 (0.93) *
EST	0.52 (0.48) *	**0.77 (0.23)**	0.29 (0.69) *	**0.94 (0.09)**	**0.67 (0.22)**	0.18 (0.65) *		-
CHE	−0.35 (0.29) *	0.32 (0.37) *	**−0.73 (0.18)**	**−0.63 (0.20)**	**−0.75 (0.22)**	0.01 (0.75) *	−0.09 (0.52) *	

CZE—Czech Republic; DFS—Denmark, Finland, and Sweden; FRA—France; GBR—Great Britain; IRL—Ireland; SVN—Slovenia; EST—Estonia; CHE—Switzerland. nc—Estimation did not converge; *—correlation was not used for the construction of a full Interbeef matrix since it was not statistically significant.

## Data Availability

The data used in this study are the property of the breeders’ organization of the participating countries and, therefore, cannot be publicly shared. The derived data supporting this study are available from the corresponding authors upon reasonable request.

## Supplementary Materials

The following supporting information can be downloaded at https://www.mdpi.com/article/10.3390/genes15020216/s1: Table S1. Definition of calving ease scoring according to country; Table S2. Size of performance datasets (above diagonal) and pedigrees (below diagonal) for pairwise variance components estimation for birth weight; Table S3. Size of performance datasets (above diagonal) and pedigrees (below diagonal) for pairwise variance components estimation for calving ease; Table S4. Weights used for the bending of the full Interbeef correlation matrix; Table S5. Full Interbeef genetic correlation matrix before bending; Table S6. Full Interbeef matrix after bending: genetic correlations (below diagonal), genetic variances (diagonal), and genetic covariances (above diagonal); Table S7. Deviations of the genetic correlations after bending.
