# Supplementary material for: Genetic Parameters for Limousine Interbeef Genetic Evaluation of Calving Traits
Source: Genes (Basel). 2024 Feb 7;15(2):216. doi: 10.3390/genes15020216 (PMC10887883; doi:10.3390/genes15020216)
Supplement: Supplementary file 1 [file genes-15-00216-s001.zip › genes-2839558-supplementary.pdf]

Genetic parameters for Limousine Interbeef genetic evaluation for calving traits

Zdeňka Veselá [1,*], Michaela Brzáková [1], Alexandra Novotná [1], Luboš Vostrý [1, 2]

[1] Department of Genetics and Breeding of Farm Animals, Institute of Animal Science, 104 00 Prague, Czech Republic; mbrzakova781@gmail.com (M.B.); novotna.alexandra@vuzv.cz (A.N.); vostry@af.czu.cz (L.V.)
[2] Faculty of Agrobiology, Food and Natural Resources, Czech University of Life Sciences Prague, 165 00 Prague, Czech Republic
* Correspondence: vesela.zdenka@vuzv.cz

Supplementary Materials: Supplementary Tables S1, S2, S3, S4, S5, S6, S7

Supplementary Table S1. Definition of calving ease scoring according to country.

Population	Score				
	**1 / 100(CHE)**	**2 / 200 (CHE)**	**3 / 300 (CHE)**	**4**	**5**
CZE	no assistance	easy pull	hard pull	c.s.	
EST	no assistance	→	vet assistance		
FRA	easy	→	→	→	embryotomy
GBR	no assistance	easy pull	hard pull	vet assistance	c.s.
IRL	no assistance	easy pull	hard pull	vet assistance	
SVN	easy	→	→	→	embryotomy
CHE	hard pull + c.s.	easy pull	no assistance		

CZE–Czech Republic; EST–Estonia; FRA–France; GBR–Great Britain; IRL–Ireland; SVN–Slovenia; CHE–Switzerland

c.s. – cesarean section

Supplementary Table S2. Size of performance data-sets (above diagonal) and pedigrees (below diagonal) for pairwise variance components estimation for birth weight.

	**CZE**	**DFS**	**FRA**	**GBR**	**IRL**	**SVN**	**CHE**
**CZE**		51,795	43,161	30,025	23,445	17,510	64,932
**DFS**	92,008		72,247	48,405	30,488	24,763	88,726
**FRA**	132,290	159,013		43,348	36,707	39,673	134,695
**GBR**	65,055	99,582	132,250		33,745	27,799	59,079
**IRL**	50,397	65,557	116,218	75,013		8,024	46,642
**SVN**	30,855	48,207	88,907	61,759	22,228		32,731
**CHE**	38,695	52,995	52,444	28,648	22,599	18,699	

CZE–Czech Republic; DFS–Denmark and Finland and Sweden; FRA–France; GBR–Great Britain; IRL–Ireland; SVN–Slovenia; CHE–Switzerland

Supplementary Table S3. Size of performance data-sets (above diagonal) and pedigrees (below diagonal) for pairwise variance components estimation for calving ease.

	**CZE**	**DFS**	**FRA**	**GBR**	**IRL**	**SVN**	**EST**	**CHE**
**CZE**		60,212	42,538	35,130	41,634	15,241	23,356	27,346
**DFS**	108,783		70,533	51,979	56,259	22,983	51,159	35,756
**FRA**	132,105	174,020		41,642	76,128	39,497	37,737	52,241
**GBR**	69,340	112,947	131,243		60,073	33,570	37,890	27,237
**IRL**	97,814	117,723	210,284	134,893		21,960	28,411	43,700
**SVN**	27,513	48,250	90,231	75,976	58,809		9,353	18,131
**EST**	43,350	94,440	99,722	87,349	71,127	18,871		22,953
**CHE**	49,711	67,339	134,344	58,597	98,274	32,067	43,591	

CZE–Czech Republic; DFS–Denmark and Finland and Sweden; FRA–France; GBR–Great Britain; IRL–Ireland; SVN–Slovenia; EST–Estonia; CHE–Switzerland

Supplementary Table S4. Weights used for the bending of the full Interbeef correlation matrix.

	Direct															Maternal											
	Birth weight							Calving ease								Birth weight						Calving ease					
	**CZE**	**DFS**	**FRA**	**GBR**	**ILR**	**SVN**	**CHE**	**CZE**	**DFS**	**FRA**	**GBR**	**IRL**	**SVN**	**EST**	**CHE**	**CZE**	**DFS**	**FRA**	**GBR**	**SVN**	**CHE**	**CZE**	**DFS**	**FRA**	**GBR**	**SVN**	**CHE**
**CZE**																											
**DFS**	2,560																										
**FRA**	2,080	2,860																									
**GBR**	1,700	1,820	1,700																								
**IRL**	2,280	2,180	2,340	3,340																							
**SVN**	1,000	1,280	1,520	1,000	1,000																						
**CHE**	2,380	2,500	3,020	1,740	1,760	1,240																					
**CZE**	9,999	1	1	1	1	1	1																				
**DFS**	1	1	1	1	1	1	1	3,020																			
**FRA**	1	1	9,999	1	1	1	1	6,240	5,320																		
**GBR**	1	1	1	9,999	1	1	1	3,120	3,460	9,040																	
**IRL**	1	1	1	1	9,999	1	1	2,940	3,260	5,600	7,180																
**SVN**	1	1	1	1	1	1	1	1,300	1,420	1,660	1,320	1,360															
**EST**	1	1	1	1	1	1	1	1,000	2,200	1,000	1,580	1,580	1,000														
**CHE**	1	1	1	1	1	1	9,999	1,000	1,000	4,060	2,340	2,340	1,000	1,000													
**CZE**	9,999	1	1	1	1	1	1	9,999	1	1	1	1	1	1	1												
**DFS**	1	9,999	1	1	1	1	1	1	1	1	1	1	1	1	1	500											
**FRA**	1	1	9,999	1	1	1	1	1	1	9,999	1	1	1	1	1	500	500										
**GBR**	1	1	1	9,999	1	1	1	1	1	1	1	1	1	1	1	500	500	765									
**SVN**	1	1	1	1	1	9,999	1	1	1	1	1	1	1	1	1	500	500	500	500								
**CHE**	1	1	1	1	1	1	9,999	1	1	1	1	1	1	1	9,999	500	895	500	760	580							
**CZE**	9,999	1	1	1	1	1	1	9,999	1	1	1	1	1	1	1	9,999	1	1	1	1	1						
**DFS**	1	1	1	1	1	1	1	1	9,999	1	1	1	1	1	1	1	1	1	1	1	1	500					
**FRA**	1	1	9,999	1	1	1	1	1	1	9,999	1	1	1	1	1	1	1	9,999	1	1	1	3,860	500				
**GBR**	1	1	1	1	1	1	1	1	1	1	9,999	1	1	1	1	1	1	1	1	1	1	500	500	5,920			
**SVN**	1	1	1	1	1	1	1	1	1	1	1	1	9,999	1	1	1	1	1	1	1	1	500	500	500	500		
**CHE**	1	1	1	1	1	1	9,999	1	1	1	1	1	1	1	9,999	1	1	1	1	1	9,999	500	500	500	500	500	

CZE–Czech Republic; DFS–Denmark and Finland and Sweden; FRA–France; GBR–Great Britain; IRL–Ireland; SVN–Slovenia; EST–Estonia; CHE–Switzerland

Supplementary Table S5. Full Interbeef genetic correlation matrix before bending.

	Direct															Maternal											
	Birth weight							Calving ease								Birth weight						Calving ease					
	CZE	DFS	FRA	GBR	ILR	SVN	CHE	CZE	DFS	FRA	GBR	IRL	SVN	EST	CHE	CZE	DFS	FRA	GBR	SVN	CHE	CZE	DFS	FRA	GBR	SVN	CHE
**CZE**																											
**DFS**	0.70																										
**FRA**	0.91	0.88																									
**GBR**	0.82	0.75	0.81																								
**IRL**	0.82	0.88	0.85	0.87																							
**SVN**	0.82	0.91	0.87	0.87	0.85																						
**CHE**	0.68	0.96	0.85	0.81	0.94	0.84																					
**CZE**	**0.25**	0	0	0	0	0	0																				
**DFS**	0	0	0	0	0	0	0	0.83																			
**FRA**	0	0	**0.69**	0	0	0	0	0.62	0.70																		
**GBR**	0	0	0	**0.53**	0	0	0	0.75	0.82	0.73																	
**IRL**	0	0	0	0	**0.62**	0	0	0.78	0.78	0.80	0.65																
**SVN**	0	0	0	0	0	0	0	0.76	0.77	0.82	0.67	0.86															
**EST**	0	0	0	0	0	0	0	0.75	0.77	0.75	0.94	0.67	0.75														
**CHE**	0	0	0	0	0	0	**−0.63**	−0.75	−0.75	−0.73	−0.63	−0.75	−0.75	−0.75													
**CZE**	**−0.48**	0	0	0	0	0	0	**−0.01**	0	0	0	0	0	0	0												
**DFS**	0	**−0.15**	0	0	0	0	0	0	0	0	0	0	0	0	0	0.60											
**FRA**	0	0	**−0.61**	0	0	0	0	0	0	**−0.20**	0	0	0	0	0	0.60	0.60										
**GBR**	0	0	0	**−0.37**	0	0	0	0	0	0	0	0	0	0	0	0.60	0.60	0.41									
**SVN**	0	0	0	0	0	**−0.49**	0	0	0	0	0	0	0	0	0	0.60	0.60	0.60	0.60								
**CHE**	0	0	0	0	0	0	**−0.72**	0	0	0	0	0	0	0	**0.39**	0.60	0.59	0.60	0.58	0.70							
**CZE**	**0.04**	0	0	0	0	0	0	**−0.47**	0	0	0	0	0	0	0	**0.42**	0	0	0	0	0						
**DFS**	0	0	0	0	0	0	0	0	**−0.20**	0	0	0	0	0	0	0	0	0	0	0	0	0.60					
**FRA**	0	0	**−0.45**	0	0	0	0	0	0	**−0.56**	0	0	0	0	0	0	0	**0.28**	0	0	0	0.56	0.60				
**GBR**	0	0	0	0	0	0	0	0	0	0	**−0.35**	0	0	0	0	0	0	0	0	0	0	0.60	0.60	0.67			
**SVN**	0	0	0	0	0	0	0	0	0	0	0	0	**−0.51**	0	0	0	0	0	0	0	0	0.60	0.60	0.60	0.60		
**CHE**	0	0	0	0	0	0	**0.24**	0	0	0	0	0	0	0	**−0.53**	0	0	0	0	0	**−0.24**	−0.60	−0.60	−0.60	−0.60	−0.60	

CZE–Czech Republic; DFS–Denmark and Finland and Sweden; FRA–France; GBR–Great Britain; IRL–Ireland; SVN–Slovenia; EST–Estonia; CHE–Switzerland

Supplementary Table S6. Full Interbeef matrix after bending: genetic correlations (below diagonal), genetic variances (diagonal), and genetic covariances (above diagonal).

	Direct															Maternal											
	Birth weight							Calving ease								Birth weight						Calving ease					
	CZE	DFS	FRA	GBR	ILR	SVN	CHE	CZE	DFS	FRA	GBR	IRL	SVN	EST	CHE	CZE	DFS	FRA	GBR	SVN	CHE	CZE	DFS	FRA	GBR	SVN	CHE
**CZE**	4.10	4.01	3.42	2.79	4.08	4.68	4.35	0.06	0.01	0.04	0.08	0.15	0.01	0.00	−5.32	−0.89	−0.04	−0.65	−0.10	−0.36	−0.40	0.00	0.01	0.00	0.02	0.01	−2.20
**DFS**	0.67	8.77	5.18	3.98	6.49	7.94	8.68	0.00	−0.04	0.05	0.03	0.14	−0.04	−0.02	−11.37	−0.02	−0.67	−0.62	0.12	−0.26	−1.39	0.02	0.00	−0.01	0.02	0.00	0.14
**FRA**	0.76	0.78	4.98	2.89	4.53	5.39	5.74	0.05	0.03	0.09	0.12	0.22	0.06	0.01	−12.53	−0.40	−0.30	−1.32	−0.13	−0.68	−0.83	0.00	0.00	−0.03	−0.02	−0.01	0.68
**GBR**	0.73	0.71	0.68	3.60	4.11	4.51	4.50	0.05	0.02	0.04	0.19	0.16	0.01	0.02	−7.35	−0.17	0.02	−0.22	−0.56	−0.28	−0.51	0.01	0.00	0.00	−0.04	0.01	−1.17
**IRL**	0.74	0.81	0.75	0.80	7.31	6.46	7.27	0.08	0.03	0.07	0.14	0.44	0.07	0.01	−16.00	−0.16	0.04	−0.27	−0.14	−0.05	−0.74	0.01	0.00	0.00	0.02	0.01	−1.09
**SVN**	0.69	0.80	0.72	0.71	0.71	11.26	8.11	0.01	−0.03	0.04	0.06	0.11	−0.04	−0.02	−6.60	−0.34	−0.06	−0.77	−0.13	−3.31	−1.16	0.02	0.00	−0.01	0.00	0.00	−1.20
**CHE**	0.62	0.84	0.74	0.68	0.77	0.69	12.13	0.06	0.02	0.08	0.14	0.32	0.05	0.02	−34.30	−0.45	−0.77	−0.92	−0.40	−1.35	−3.38	0.01	0.00	−0.01	0.01	0.01	10.06
**CZE**	**0.24**	0.01	0.18	0.19	0.24	0.03	0.14	0.02	0.01	0.01	0.02	0.03	0.01	0.01	−1.33	0.00	0.01	0.01	0.01	0.01	0.02	0.00	0.00	0.00	0.00	0.00	0.16
**DFS**	0.05	−0.14	0.11	0.10	0.12	−0.09	0.06	0.79	0.01	0.00	0.02	0.02	0.01	0.01	−1.15	0.01	−0.01	0.01	0.00	−0.01	0.00	0.00	0.00	0.00	0.00	0.00	0.01
**FRA**	0.28	0.24	**0.63**	0.32	0.41	0.19	0.35	0.60	0.68	0.00	0.01	0.01	0.01	0.00	−0.73	0.00	0.00	−0.01	0.00	0.01	0.00	0.00	0.00	0.00	0.00	0.00	0.09
**GBR**	0.21	0.05	0.28	**0.50**	0.27	0.10	0.20	0.70	0.77	0.70	0.04	0.04	0.02	0.01	−1.97	0.02	0.01	0.01	−0.03	0.01	0.01	0.00	0.00	0.00	−0.01	0.00	0.09
**IRL**	0.26	0.17	0.36	0.30	**0.58**	0.12	0.32	0.73	0.74	0.76	0.63	0.08	0.04	0.01	−3.26	0.02	0.01	0.02	0.01	0.04	0.00	0.00	0.00	0.00	0.00	0.00	0.07
**SVN**	0.04	−0.09	0.16	0.02	0.15	−0.07	0.08	0.66	0.70	0.71	0.59	0.74	0.03	0.01	−1.80	0.01	0.00	0.01	0.00	0.00	0.01	0.00	0.00	0.00	0.00	−0.01	0.18
**EST**	0.01	−0.11	0.10	0.14	0.04	−0.07	0.07	0.65	0.74	0.64	0.82	0.60	0.66	0.00	−0.71	0.00	0.00	0.00	0.00	0.00	0.00	0.00	0.00	0.00	0.00	0.00	0.02
**CHE**	−0.15	−0.23	−0.33	−0.23	−0.35	−0.12	**−0.58**	−0.61	−0.63	−0.68	−0.58	−0.68	−0.62	−0.63	287.3	−1.46	−0.14	−0.54	−0.42	−0.53	9.50	0.10	0.03	0.08	0.07	0.13	−106
**CZE**	**−0.47**	−0.01	−0.19	−0.10	−0.06	−0.11	−0.14	**−0.02**	0.10	0.06	0.09	0.09	0.08	0.06	−0.09	0.90	0.63	0.42	0.33	0.82	0.55	0.02	0.00	0.00	0.00	0.00	−0.54
**DFS**	−0.01	**−0.15**	−0.09	0.01	0.01	−0.01	−0.14	0.06	−0.03	0.04	0.03	0.02	−0.02	−0.02	−0.01	0.43	2.37	0.70	0.56	1.40	1.09	0.01	0.00	0.00	0.00	0.00	0.48
**FRA**	−0.31	−0.20	**−0.57**	−0.11	−0.10	−0.22	−0.25	0.08	0.05	**−0.21**	0.07	0.07	0.04	0.05	−0.03	0.43	0.44	1.08	0.29	0.91	0.62	0.00	0.00	0.01	0.00	0.00	0.51
**GBR**	−0.06	0.05	−0.07	**−0.36**	−0.06	−0.05	−0.14	0.08	0.00	0.05	−0.17	0.02	0.01	0.01	−0.03	0.43	0.44	0.34	0.67	0.73	0.55	0.00	0.00	0.00	0.00	0.00	0.11
**SVN**	−0.08	−0.04	−0.15	−0.07	−0.01	**−0.47**	−0.19	0.05	−0.03	0.04	0.03	0.06	−0.01	−0.02	−0.01	0.41	0.44	0.42	0.43	4.32	1.47	0.01	0.00	0.00	0.00	0.00	0.29
**CHE**	−0.14	−0.33	−0.26	−0.19	−0.19	−0.24	**−0.67**	0.09	0.03	0.01	0.04	0.01	0.02	0.02	**0.39**	0.40	0.49	0.41	0.46	0.49	2.09	0.00	0.00	0.00	0.00	0.00	−4.23
**CZE**	**0.03**	0.10	0.03	0.12	0.09	0.10	0.07	**−0.46**	−0.09	−0.11	−0.08	−0.08	−0.07	−0.04	0.10	**0.41**	0.07	0.02	0.06	0.08	0.05	0.00	0.00	0.00	0.00	0.00	−0.30
**DFS**	0.07	−0.02	−0.02	0.01	0.01	−0.01	0.02	−0.06	**−0.20**	−0.04	−0.04	0.00	0.00	0.04	0.03	0.06	−0.01	0.00	0.00	0.00	−0.01	0.43	0.01	0.00	0.00	0.00	−0.42
**FRA**	−0.01	−0.11	**−0.43**	−0.04	−0.04	−0.07	−0.06	−0.09	−0.04	**−0.54**	−0.16	−0.08	−0.15	−0.01	0.13	0.10	−0.03	**0.28**	−0.04	−0.02	−0.05	0.53	0.43	0.00	0.00	0.00	−0.19
**GBR**	0.08	0.04	−0.07	−0.16	0.05	0.00	0.03	−0.05	−0.03	−0.17	**−0.34**	0.05	−0.01	−0.05	0.03	0.03	0.01	−0.02	0.01	−0.01	−0.02	0.44	0.45	0.66	0.02	0.01	−0.76
**SVN**	0.03	0.01	−0.03	0.04	0.03	0.00	0.02	−0.05	0.02	−0.14	−0.03	−0.05	**−0.50**	0.02	0.09	0.03	0.00	−0.02	0.01	0.01	−0.01	0.25	0.43	0.41	0.43	0.01	−0.44
**CHE**	−0.09	0.00	0.02	−0.05	−0.03	−0.03	**0.24**	0.10	0.01	0.12	0.04	0.02	0.09	0.02	**−0.51**	−0.05	0.03	0.04	0.01	0.01	**−0.24**	−0.42	−0.44	−0.42	−0.44	−0.42	149.4

CZE–Czech Republic; DFS–Denmark and Finland and Sweden; FRA–France; GBR–Great Britain; IRL–Ireland; SVN–Slovenia; EST–Estonia; CHE–Switzerland

Supplementary Table S7. Differences of the genetic correlations after bending.

	**Direct**															**Maternal**											
	**Birth weight**							**Calving ease**								**Birth weight**						**Calving ease**					
	**CZE**	**DFS**	**FRA**	**GBR**	**ILR**	**SVN**	**CHE**	**CZE**	**DFS**	**FRA**	**GBR**	**IRL**	**SVN**	**EST**	**CHE**	**CZE**	**DFS**	**FRA**	**GBR**	**SVN**	**CHE**	**CZE**	**DFS**	**FRA**	**GBR**	**SVN**	**CHE**
**CZE**																											
**DFS**	−0.02																										
**FRA**	−0.11	−0.09																									
**GBR**	−0.08	−0.07	−0.13																								
**IRL**	−0.03	−0.06	−0.08	−0.08																							
**SVN**	−0.11	−0.14	−0.15	−0.18	−0.14																						
**CHE**	−0.05	−0.13	−0.10	−0.14	−0.14	−0.14																					
**CZE**	**−0.02**	0.01	0.18	0.19	0.24	0.03	0.14																				
**DFS**	0.05	−0.14	0.11	0.10	0.12	−0.09	0.06	−0.04																			
**FRA**	0.28	0.24	**−0.06**	0.32	0.41	0.19	0.35	−0.02	−0.03																		
**GBR**	0.21	0.05	0.28	**−0.03**	0.27	0.10	0.20	−0.05	−0.05	−0.03																	
**IRL**	0.26	0.17	0.36	0.30	**−0.04**	0.12	0.32	−0.05	−0.04	−0.04	−0.02																
**SVN**	0.04	−0.09	0.16	0.02	0.15	−0.07	0.08	−0.10	−0.07	−0.11	−0.08	−0.12															
**EST**	0.01	−0.11	0.10	0.14	0.04	−0.08	0.07	−0.11	−0.03	−0.12	−0.12	−0.07	−0.10														
**CHE**	−0.16	−0.23	−0.33	−0.23	−0.35	−0.12	**0.05**	0.15	0.13	0.05	0.05	0.07	0.14	0.13													
**CZE**	**0.02**	−0.01	−0.19	−0.10	−0.06	−0.11	−0.14	**−0.01**	0.10	0.06	0.09	0.09	0.08	0.06	−0.09												
**DFS**	−0.01	**0.00**	−0.09	0.01	0.01	−0.01	−0.14	0.06	−0.03	0.04	0.03	0.02	−0.02	−0.03	−0.01	−0.17											
**FRA**	−0.31	−0.20	**0.04**	−0.11	−0.10	−0.22	−0.26	0.08	0.05	**−0.01**	0.07	0.07	0.04	0.05	−0.03	−0.17	−0.16										
**GBR**	−0.06	0.05	−0.07	**0.01**	−0.06	−0.05	−0.14	0.08	0.00	0.05	−0.17	0.02	0.01	0.01	−0.03	−0.17	−0.16	−0.07									
**SVN**	−0.09	−0.04	−0.15	−0.07	−0.01	**0.02**	−0.19	0.06	−0.03	0.04	0.03	0.06	−0.01	−0.02	−0.02	−0.19	−0.16	−0.18	−0.17								
**CHE**	−0.14	−0.33	−0.26	−0.19	−0.19	−0.24	**0.05**	0.09	0.03	0.01	0.04	0.01	0.02	0.02	**0.00**	−0.20	−0.10	−0.19	−0.12	−0.21							
**CZE**	**−0.01**	0.10	0.03	0.12	0.09	0.10	0.07	**0.02**	−0.09	−0.11	−0.08	−0.08	−0.07	−0.05	0.10	**−0.01**	0.07	0.02	0.06	0.08	0.05						
**DFS**	0.07	−0.02	−0.02	0.01	0.01	−0.01	0.02	−0.06	**0.00**	−0.04	−0.04	0.00	0.00	0.05	0.03	0.06	−0.01	0.00	−0.01	0.00	−0.01	−0.17					
**FRA**	−0.01	−0.11	**0.02**	−0.04	−0.04	−0.07	−0.06	−0.09	−0.04	**0.02**	−0.16	−0.08	−0.15	−0.01	0.13	0.10	−0.03	**0.00**	−0.04	−0.03	−0.06	−0.03	−0.17				
**GBR**	0.08	0.04	−0.07	−0.17	0.05	0.00	0.03	−0.05	−0.03	−0.17	**0.01**	0.05	−0.01	−0.05	0.03	0.03	0.01	−0.02	0.01	−0.01	−0.02	−0.16	−0.15	−0.02			
**SVN**	0.04	0.01	−0.03	0.04	0.03	0.00	0.02	−0.05	0.02	−0.14	−0.03	−0.05	**0.01**	0.03	0.09	0.03	0.00	−0.02	0.01	0.01	−0.02	−0.07	−0.17	−0.19	−0.17		
**CHE**	−0.09	0.00	0.03	−0.05	−0.03	−0.03	**0.00**	0.10	0.01	0.12	0.04	0.02	0.09	0.02	**0.02**	−0.05	0.03	0.04	0.01	0.01	**0.00**	0.18	0.16	0.18	0.16	0.18	

CZE–Czech Republic; DFS–Denmark and Finland and Sweden; FRA–France; GBR–Great Britain; IRL–Ireland; SVN–Slovenia; EST–Estonia; CHE–Switzerland
